# Serum Uric Acid – Risk Factor for Acute Ischemic Stroke and Poor Outcomes

**DOI:** 10.7759/cureus.6007

**Published:** 2019-10-28

**Authors:** Muhammad Ali Tariq, Sohaib A Shamim, Kiran F Rana, Aisha Saeed, Bilal Haider Malik

**Affiliations:** 1 Internal Medicine, California Institute of Behavioral Neurosciences and Psychology, Fairfield, USA; 2 Neurology, California Institute of Behavioral Neurosciences and Psychology, Fairfield, USA; 3 Family Medicine, California Institute of Behavioral Neurosciences and Psychology, Fairfield, USA

**Keywords:** serum uric acid levels, ischemic stroke, uric acid, hyperuricemia, stroke

## Abstract

Over the last decades several studies among the adult population have attempted to establish a correlation between the risk of stroke incidence and serum uric acid (SUA) concentration, and how these levels influence the patient’s neurological outcome after a stroke. But, to date, the results are conflicting. In this review, an extensive literature search was performed through PubMed for articles published until May 2019 to review the association. The study selection was narrowed by searching PubMed database using the Medical Subject Headings (MesH) and associated keywords. Only articles conducted in English and on human subjects were included. We considered an article for this review if it had statistics on either the incidence, stroke mortality or post-stroke functional outcomes along with serum uric acid levels in adults.

This review includes 21 articles with data of 33,580 cases of stroke and 1,100,888 participants. We can divide the articles reviewed into two separate cohorts of studies. One relates serum uric acid levels to stroke frequency and mortality, while the other is associated with serum uric acid and outcomes for stroke survivors. Based on our review, no significant relationship is observed with uric acid exhibiting protective effects on stroke outcome. Large clinical trials are advised to provide well-defined solutions to further assess the benefits of uric acid level lowering treatment in patients of vascular events, such as a stroke. However, we confidently report that increasing uric acid levels poses a higher risk for incidence of stroke.

## Introduction and background

Stroke is the fifth most prevalent cause of death in the United States with an annual reported figure of 142,000 deaths while 795,000 individuals experience a new or recurrent stroke. In addition, stroke has also contributed as a leading cause of long-term disability. The estimated annual expense of treating stroke amounts to $34 billion for the United States government [1].

Uric acid is a weak organic acid that is the end catabolite of purine nucleotide metabolism. Uric acid stands out as one of the most essential antioxidants in blood with a concentration almost tenfold greater than other antioxidants. It functions as a scavenger for superoxide, hydroxyl, and oxygen radicals. Hyperuricemia is described as serum uric acid concentration being greater than 6.8 mg/dL [2]. Uric acid levels regulate in the body by the equilibrium created between its production and discharge by the kidney. Uric acid is either produced from exogenous sources of high purine intake such as meat, seafood, animal organs or from endogenous sources like tissue catabolism and de novo synthesis of purines from RNA and DNA bases [3]. Hyperuricemia is involved in the pathophysiology of several diseases such as gout, chronic kidney disease, cardiovascular diseases such as coronary heart disease, metabolic syndrome, obesity, hypertension and hyperlipidemia [4,5]. For decades it has been recognized as a major cause of gouty arthritis due to deposits of uric acid as monosodium urate (MSU) crystals in the first metatarsophalangeal joint and other various joints, tendons and tissues throughout the body. Renal under-excretion of uric acid is accountable for 90% cases of gout while the increased synthesis of uric acid contributes to only 10% cases [6].

However, it is questionable whether hyperuricemia is a major risk factor to the onset of stroke. During the last few decades, numerous prospective studies have analyzed the relationship of serum uric acid levels and stroke frequency, but results have been conflicting. Several studies have shown that uric acid is a free radical scavenger and antioxidant that protects the brain from oxidative damage, thereby preventing worse post-stroke neurological outcomes [7,8]. While several other studies have shown that hyperuricemia is responsible for increase of stroke incidence and mortality [9-11]. This disparity is due to the variations in sample size, attributes of the representative population such as geographic differences, ethnicity, gender, age, socio-economic factors and the methods used to conduct the study.

The present scientific literature is therefore reviewed to address this disparity and to provide an exhaustive estimate of the possible relationship between serum uric acid, stroke incident, stroke mortality, and to verify whether hyperuricemia is a risk factor for stroke. Our study may arouse the interest of many experts, thereby providing a foundation for further academic studies and clinical trials to eventually offer better management and care in events of stroke.

## Review

Literature search

A literature search of the PubMed electronic databases was conducted until May 2019 for studies related with stroke incidence and serum uric acid levels. Bibliography and citations from the retrieved articles were searched for further references. Only articles in English were considered, and the search was restricted to human studies. If any similarity arose in the patient cohort in different publications, we selected only latest article for review. We did not use Meta-Analysis of Observational Studies in Epidemiology (MOOSE) guidelines and the Preferred Reporting Items for Systematic Reviews and Meta-Analysis (PRISMA) checklist.

We performed the preliminary search from PubMed using simple keyword and MESH keywords, as mentioned in Table 1. The number of articles obtained from the search are mentioned in Table 2 and Table 3. Therefore, we identified 1265 articles from the initial database search. After the first scan based on titles and abstracts, we excluded 1130 articles for one of the following reasons: 1) It did not relate uric acid to primary objectives of our review article, 2) it was a review article, editorial, letter to editor, abstract not available, 3) it was an animal study, 4) it was not a cohort study. A potential of 135 studies were retained for further evaluation. On further analysis in accordance with the pre-determined inclusion and exclusion criteria, only 21 articles were included in this review.

**Table 1:** Detail Search Strategy MeSH: Medical Subject Headings

1. MeSH term search	2. Simple keyword search
Uric acid	Cerebrovascular disorders
Hyperuricemia	Urate
Stroke	ischemic stroke
Brain ischemia	Cerebral infraction

**Table 2:** Search Results with MeSH Keywords MeSH: Medical Subject Headings

MeSH keyword search	Number of results
Uric acid and stroke	175
Brain ischemia and uric acid	138
Hyperuricemia and stroke	44
Hyperuricemia and brain ischemia	18

**Table 3:** Search Results with Simple Keywords

Simple keywords search	Number of results
Urate and cerebrovascular events	568
Ischemic stroke and urate	233
Cerebral infarction and urate	89

Study selection and data collection

The articles included for review must comply with the predetermined inclusion and exclusion criteria. Articles that fulfilled the following inclusion criteria must be: case control or cohort studies with a follow-up period either equal or greater than three months/90 days, a sample size of at least 100 participants with availability of baseline uric acid levels. We excluded articles from the initial search of database in according to the exclusion criteria which included: animal studies, letters to editors, reviews articles, editorial comments, cross-sectional studies, studies involving multiple physiological systems and studies published in non-English language. The following key details were obtained from each study included in the review article: the first author’s name, year of publication, country of origin of the study, total sample size for the study, percentage of the male in the sample size, average age of participants, time period of follow-up, the number of stroke incidents and outcomes of such incidents.

Results

Study Characteristics

Twenty-one studies constituting data from 1,100,888 people are included in the review. Since the UK-TIA Aspirin trial study and the Oxford TIA study are reported separately in the article by Koton et al. hence they are considered two separate studies [12]. Among the 21 studies, two were from the United States and two were from the United Kingdom. Five studies represented data from China and three from Japan. Six were from other European countries, one each from Israel, Singapore, Korea and Africa. The number of participants ranged from 140 in a study by Newman et al. to 417,734 in AMORIS study by Holme et al. [13,14]. The Follow-up period extended between three months to 23 years. In three studies the sample size only consisted of men [11,15,16]. While two studies represented data from women only [17,18]. In our observation of the studies included in our literature review, the results can be divided into two broader classifications. One for the uric acid levels and stroke incidence while other discussed the uric acid levels in stroke survivors, its impact on the functional outcome.

Uric Acid and Incidence of Strokes

Table 4 displays the key attributes of the studies where the outcomes of interest were incidences of strokes. The events of stroke were defined using the medical records and International Classification of Diseases (ICD) codes from hospital records or death certificates. We can further classify results in this classification into two groups. Firstly, studies where no association between serum acid levels and stroke could be established. Secondly, studies where uric acid level predicted a positive correlation with the incidence of stroke. It was not possible to establish a role of serum uric acid in stroke in studies conducted by Koton et al., Jee et al., Sakata et al. [12,15,19]. Although, it can be observed in the study by Jee et al. that quintiles 3 (307-354 mmol/l), quintiles 4 (355-414 mmol/l) and quintiles 5 (>414 mmol/l) with higher serum uric acid exhibited increased risks of total stroke mortality (with 73 events, 82 events, 79 events of stroke) when compared with quintile 1 (<265 mmol/l with 84 events) but were not significant enough to be considered as an independent risk factor for death [19]. Six studies conveyed a strong positive association with uric acid levels [9,10,14,16,17,20]. A recent study published in 2019 reported elderly hyperuremic patients have a twofold increased risk of stroke [20]. This is in line with previously reported literature for example the TROMSO study of 5700 individuals over the follow-up period of 12.5 years concluding an 87 μmol/L increase in serum uric acid is associated with greater prospects by almost 31% for ischemic stroke [10]. Likewise, Jimenez et al. in a female exclusive study in the United States reported each 1 mg/dL increase in uric acid concentration was connected with a 15% greater risk of ischemic stroke [17]. Interestingly, handful studies illustrated that not only high uric acid levels can be dangerous, but low serum uric acid levels can also be proven to be detrimental hence predicting a U-shaped graph [11,21]. Kuo et al. reported, high (>0.66 mmol/l) or low(<0.17 mmol/L) serum uric acid levels are more likely to be a cause of increased stroke mortality while levels ranging from 0.30 to 0.41 mmol/L had lowest probability of deaths [21].

**Table 4:** Uric Acid Level and Stroke Incidences UA: Uric Acid; SUA: Serum Uric Acid

Author, year	County	Sample size	Age in years (mean/range)		% Men	Follow-up years		Outcomes		Conclusions
Tu et al. 2019 [20]	China	3243	70.8	55.0		2.9		146	High UA increased risk of stroke in older population		
Jimenez et al. 2016 [17]	United States	920	30-55	0.0		17.0		460	UA is not an independent risk factor for stroke in women but higher levels do increase the risk for stroke.		
Kamei et al. 2016 [9]	Japan	155,322	40-73	39.0		2.0	2081		UA is associated with increased incidence of nonfatal stroke		
Kuo et al. 2013 [21]	China	354,110	49.8	55.0		4.6	2412		Individuals with either high or low SUA levels are at greater risk for mortality.		
Storhaug et al. 2013 [10]	Norway	5700	>25	42		12.5	430		SUA positively associated with increased risk for ischemic stroke in men.		
Holme et al. 2009 [14]	Sweden	417,734	30-85	53		11.8	16276		Increase in UA associated with increased risk of ischemic stroke.		
Strasak et al. 2008 [18]	Austria	28,613	62.6	0.0		15.2	776		In elderly post-menopausal women SUA is an independent predictor of death from stroke.		
Strasak et al. 2008 [16]	Austria	83,683	41.6	100		13.6	645		Hyperuricemia is associated with an increased risk of death due to stroke.		
Koton et al. 2008 [12]	United Kingdom	1842-Aspirin Trial 289-Oxford Trial	60.0, 69.0	72.1, 61.7		7.25, 10	214, 45		SUA failed to predict risk or severity of stroke.		
Gerber et al. 2006 [11]	Israel	9125	49.0	100		23	292		Hyperuricemia increased mortality risk. Additionally association between low SUA levels and fatal stroke was established.		
Jee et al. 2004 [15]	Korea	22,698	30-77	100		9.0	192		UA levels are not an independent risk factor of mortality due to stroke.		
Sakata et al. 2001 [19]	Japan	8172	>30	44.0		14.0	174		SUA levels are not associated with increased risk of death due to stroke.		

Effect of Uric Acid on Outcomes of Stroke Survivors

Table 5 lists the studies where the primary outcome was the occurrence of post-stroke poor outcomes. Poor outcomes were defined as death or poor functional neurological outcomes, as defined by Modified Rankin Scale (mRS) with a score more than 2 (evaluated at hospital discharge, 30 days, 90 days or one year after stroke onset). In our review, we noted three separate trends of results. A select few studies found a positive association between high uric acid levels and good outcomes [22-23]. The study of Amaro et al. in Spain found 317 patients noted an improved recovery with higher serum uric acid levels when co-treated with reperfusion therapy [22]. Similarly, Wang et al. reported that high serum uric acid levels at hospital admission were especially protective in men [23]. Four of studies reported data of poor outcome after stroke due to increase serum uric acid levels [24-27]. Weir et al. studied around 2500 stroke survivors, indicating that the addition of 1.7 mg/dl in urate level was associated with an increased risk of stroke by 27% anticipating a worse outcome and increased chances of repeated stroke [26]. More recently, the analysis by Mapoure et al. confirmed the earlier results by reporting that higher serum uric acid quintile ranges (SUA 71-84 mg/L and SUA > 85 mg/L) had more proportion of deaths and were statistically more likely to correspond with poorer outcomes [27]. However, a series of interesting results were also found in three studies where no conclusive relation could be identified between the outcome of stroke and uric acid levels [7,28,29]. Miedema et al. analyzed blood samples among 226 patients of stroke and reported no link between serum uric acid and probability of acute ischemic stroke at either short or long-term follow-up [28]. Research by Yang et al. on the functional outcome of 710 patients within three months of ischemic stroke reported the findings indicating a U shaped nonlinear relationship. Thus, serum uric acid may exhibit protective or detrimental effects depending on its concentration [29]. This in consistent with a previously reported study by Seet et al. which also observed a nonlinear relationship between uric acid and outcome one year after stroke, and documented the best functional outcome for patients with serum uric acid concentrations of 0.28 mmol/L to 0.32 mmol/L [7].

**Table 5:** Uric Acid Levels and Occurrence of Poor Outcome and Deaths in Stroke Survivors UA: Uric Acid; SUA: Serum Uric Acid

Author, Year	Country	Sample size (patients with ischemic stroke)	Age in years (Mean/Range)	% men	Follow-up months	Outcomes	Conclusion
Wang et al. 2018 [23]	China	1166	64.5	62.7	12	35 - deaths, 294 - poor outcome	Association between high SUA and good outcome in males.
Yang et al. 2018 [29]	China	710	59.0	52.8	3	84 - deaths, 219 - poor outcome	Association between high or low SUA levels and poor outcome.
Mapoure et al. 2017 [27]	Africa	480	62.8	53.1	3	101 - deaths, 93 - poor outcome	Association between high SUA and poor outcome.
Wu et al. 2014 [24]	China	1452	63	64.9	12	144 - deaths, 370 - poor outcome	Association between lower UA and poor outcomes
Miedema et al. 2012 [28]	Canada, United States	226	71.0	54.0	3	42 - Good outcome, 47 - Poor outcome	No association
Amaro et al. 2011 [22]	Spain	317	72.0	56.0	3	101 - Deaths, 36 - Good outcome	Association between higher UA and good outcome
Seet et al. 2009 [7]	Singapore	503	63.0	61.0	12	127 poor outcome	Association between low or high UA and poor outcome
Dawson et al. 2009 [25]	Germany	852	68	61.5	3	141 - Deaths, 434 - Poor outcome	Association between higher UA and poor outcome
Weir et al. 2003 [26]	United Kingdom	3731	72.0	48.0	3	2361 - good outcome, 333 - poor outcome	Association between higher UA and poor outcome

Discussion

Biochemistry of Uric Acid

Uric acid is a weak organic acid with a molar mass of 168.112 g/mol. Most uric acid at normal blood pH circulates as urate, a negatively charged weak salt derived from uric acid. As the terminal product of purine catabolism process, uric acid is eliminated from the human body via urine as long as renal function is not debilitated. The acceptable range of uric acid in human blood may vary in the two genders with 1.5 to 6.0 mg/dL in females and 2.5 to 7.0 mg/dL in males. A potential explanation for this difference is the influence of estrogen on the enhancement of uric acid excretion in females along with suppression of URAT1 transporter in the proximal tubule [30]. An increase in urate concentration in blood beyond the solubility limit of 6.8 mg/dl increases the risk of uric acid crystal formation known as monosodium urate [31]. Early stages of hominoid evolution lead to the functional mutation of UOX gene, which codes for the enzyme urate oxidase. Humans therefore lack the ability to efficiently excrete uric acid via kidney due to lack of conversion of uric acid to more polar compounds like allantoic acid and ammonia. The deficiency of urate oxidase with significant reabsorption of filtered urate by renal glomerulus leads to an increase in human serum uric acid levels compared to other mammals [31].

**Figure 1:**
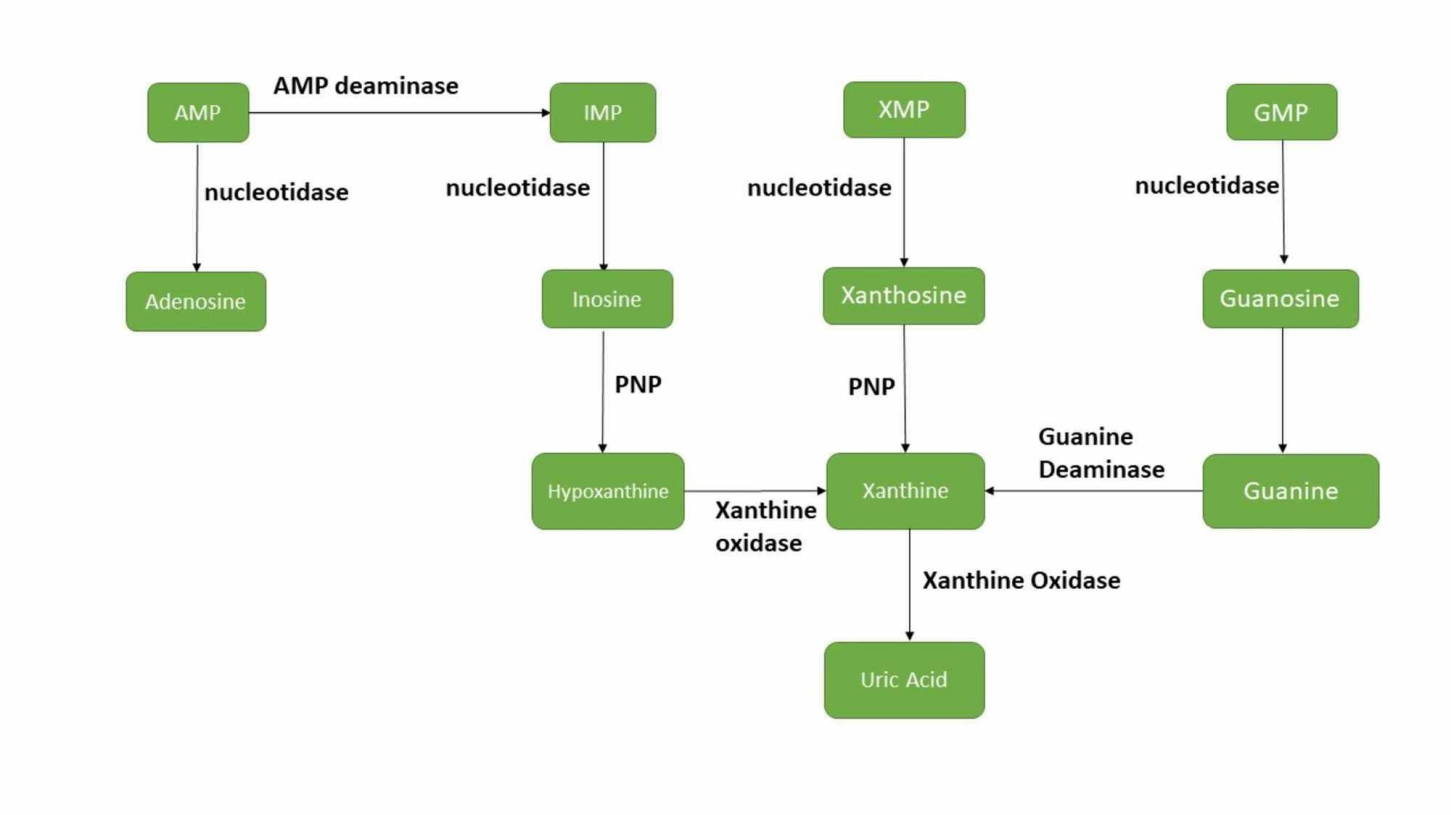
Uric Acid Metabolism AMP: Adenosine monophosphate; GMP: Guanosine monophosphate; IMP: Inosine monophosphate; PNP: Purine nucleoside phosphorylase; XMP: Xanthosine monophosphate.

Purine metabolism involves catabolism of purine nucleotides: Guanosine monophosphate (GMP), inosine monophosphate (IMP) and Adenosine monophosphate (AMP) through enzymatic processes of deamination and dephosphorylation to generate inosine, xanthosine and guanosine nucleosides. These nucleosides are then further converted via purine nucleoside phosphorylase (PNP) enzyme into purine bases hypoxanthine, xanthine and guanine, respectively. Meanwhile, the xanthine oxidoreductase (XOR) enzyme also known as Xanthine oxidase (XAO) catalyzes oxidation of hypoxanthine to xanthine while the guanine deaminase enzyme catalyzes deamination of guanine to xanthine. Finally, xanthine is irreversibly oxidized by the enzyme XOR to form uric acid, the terminal metabolite [32]. The entire pathway will be better understood with the aid of Figure 1.

Neuroprotective Effects

Uric acid is a potent antioxidant and an effective scavenger of singlet oxygen and free radicals. It provides about 60% of the free-radical scavenging capacity of the blood, with its concentration increasing significantly during stroke. Under normal physiological conditions, the antioxidant effect of uric acid is comparable or greater to ascorbate, another important antioxidant in plasma [33]. Ischemic stroke is a condition of oxidative stress in which a thrombus occludes a brain vessel which initiates an ischemic cascade. The infarct core is a region of brain deprived of blood supply and perfusion so neural tissues and cells here get irreversibly damaged. Infarct core is surrounded by ischemic penumbra, an under-perfused area comprising of functionally damaged but viable tissue, making it the ideal target site for neuroprotective therapies [34]. At the moment the most effective therapy for stroke includes recanalization and reperfusion of thrombosed arteries with a thrombolytic agent like a recombinant tissue plasminogen activator (rtPA) [35].

Animal studies have shown that administration of uric acid or uric acid analogs protects the brain against ischemic damage by minimizing infarct size and neurofunctional disability [36]. A study by Romanos et al. questioned the benefits of combined treatment of uric acid with recombinant tissue plasminogen activator (rtPA). The findings favored the combined treatment, which demonstrated reduced post-ischemic brain neutrophil infiltration, cerebral edema and infarct size [37]. The observed neuroprotective effects of uric acid led to clinical trials on humans. However, a combination of both favorable and unfavorable results are reported in humans. A pilot experiment for treatment of stroke patients with rtPA demonstrated the administration of uric acid is safe along with favorable outcomes such as drop in agents of oxidative stress [38]. In a phase 2b/3 URICO-ICTUS clinical trial, 421 stroke patients initially treated with alteplase were randomly assigned either 1000 mg of uric acid or a placebo to be co-administered intravenously with alteplase infusion. The trial revealed high tolerability of uric acid in patients but the administration of uric acid along with thrombolytic therapy did not remarkably increase percentage of patients who achieved favorable follow-up three months after stroke when compared with placebo [39]. Overall, these findings are further supported by a meta-analysis published in 2016, in which 10 studies of 8,131 patients with ischemic stroke were studied [40]. The analysis demonstrated that patients presenting with elevated serum uric acid levels at the start of stroke were shown to have a favorable clinical outcome, thereby pointing to the protective nature of uric acid.

Neurotoxic Effects

There are several proposed mechanisms that may result in uric acid functioning as neurotoxic. Higher uric acid levels proliferate smooth muscle wall enhancing LDL (low density lipoprotein) oxidation, reduce endothelial nitric oxide synthase leading to endothelial dysfunction, and increase the production of platelet-derived growth factors maximizing platelet adhesion [41]. Each of these factors could potentially stimulate a cascade of coagulation, leading to thrombus formation and arterial occlusion eventually progressing to intracranial atherosclerosis. The study by Patetsios et al. confirmed the direct role of uric acid in development of atherosclerotic plaque by comparing the different sample of atherosclerotic plaques with control specimens and reported a greater presence of uric acid and xanthine oxidase in the composition of atherosclerotic plaque specimens [42]. The reason that high uric acid level is usually associated with cerebrovascular morbidity is due to inflammation. Experimental studies have shown that hyperuricemia leads to elevated levels of agents of systemic inflammation such as interleukin 6 (IL-6), tumor necrosis factor a (TNF-a) and C-reactive protein (CRP) [43]. While, newer evidence suggests that uric acid may also have the ability to induce systemic inflammation via the NF-kB signaling pathway [44]. In recent years, meta-analysis published by Kim et al. in 2009 and by Li et al. in 2013 supports the postulate of a direct association between stroke and high uric acid levels [45,46].

What the Future Holds

We could not achieve a definitive answer. Studies have pointed out at both low and high levels of uric acid being dangerous in conditions of stroke. At the same time, review of available pre-clinical data points at the imminent role of uric acid as a viable neuroprotectant. URICO-ICTUS trial, although promising, failed to replicate these results in clinical settings. Perhaps one of the drawbacks of direct extrapolation of results from animal studies to humans since uric acid metabolism and levels differ between the two species [39]. Simultaneously, URICO-ICTUS trial has provided a detailed overview of ischemic stroke to appreciate its pathophysiology which will help develop better therapies and cure in the near future. However, our study has raised some valid questions like whether uric acid treatment as pre-stroke, post-stroke or co-treatment with rTPA could be helpful and affordable to the patients. Secondly, should abnormal uric acid concentration in conditions of stroke be supplemented or completely eliminated to maintain the optimum urate levels. It is certain that to reach some plausible conclusion regarding neuroprotective effectiveness of uric acid, it deserves to be tested in a larger phase III trial.

Limitations

This review has several limitations. Firstly, the definition of hyperuricemia and poor outcomes varied across the different studies included in our review. Secondly, the level of serum uric acid was measured only on admission and did not include the changes to the levels of uric acid afterwards including the follow-up period. Thus, repeated measurements after certain period of time decided with patient consent are therefore imperative to precisely notice the changes in uric acid levels over time and their association with stroke outcomes. Thirdly, the role of serum uric acid may vary in different stages of stroke, but the lack of data in the included studies has made it difficult to analyze this role. Fourthly, commonly used medications such as aspirin and diuretics influence serum uric acid levels, but there is no information on this in any of the studies and they are not acknowledged as confounding factors. Hence, the presence of unacknowledged or poorly measured confounders could therefore disprove the association established here in this review article. Finally, there is a possibility of language bias. Only articles in English were searched in the PubMed database for review, therefore articles in non-English languages may not appear in the search results.

## Conclusions

The objective of our review was to verify in the light of current medical literature, the association between uric acid and ischemic stroke, and whether uric acid lowering treatments would prove beneficial. Based on our review article, we can conclude that insufficient evidence presents to support the hypothesis regarding the neuroprotective effect of uric acid in conditions of ischemic stroke. Further studies and clinical trials are therefore recommended. Another conclusion drawn from our review is that high uric acid levels negatively impact the physical well-being of individuals by activating a cycle of events involving inflammatory and oxidative mechanisms of action which ultimately lead to stroke. We can therefore report that high uric acid levels or hyperuricemia are associated with an increased incidence of stroke and are a strong indicator of negative post stroke functional outcome.
